# Nanotechnology Meets Immunotherapy: Crosstalks Against Cancer

**DOI:** 10.1002/iid3.70437

**Published:** 2026-05-07

**Authors:** Nima Javanmehr, Asal Moazzami Ashtyani, Robabehbeygom Ghafelehbashi, Fateme Mousavi, Hossein Teimouri

**Affiliations:** ^1^ Student Research Committee Babol University of Medical Sciences Babol Iran; ^2^ Department of Medical Laboratory Sciences, Faculty of Allied Medicine Bam University of Medical Sciences, Bam Kerman Iran; ^3^ Department of Medical Nanotechnology, Faculty of Advanced Technologies in Medicine Iran University of Medical Sciences Tehran Iran; ^4^ Shahid Rajaei Hospital Qazvin University of Medical Sciences Qazvin Iran; ^5^ Medical Microbiology Research Center Qazvin University of Medical Sciences Qazvin Iran

**Keywords:** cancer immunotherapy, combination therapy, nanoparticles, nanotechnology, tumor microenvironment, tumor microenvironment

## Abstract

**Background:**

The convergence of nanotechnology and immunotherapy has ushered in a transformative era in cancer treatment, offering new strategies to overcome pharmacokinetic limitations and immune evasion associated with conventional therapies. While immunotherapy, spanning checkpoint inhibitors, adoptive cell transfer, and cancer vaccines, has revolutionized oncology, its efficacy remains constrained by the immunosuppressive tumor microenvironment (TME), off‐target toxicity, and poor biodistribution of therapeutic agents.

**Objective:**

This review elucidates how engineered nanoparticles (NPs) are redefining immune‐oncology by enabling the precise delivery of immunomodulators, antigens, and genetic payloads to target cells, while reprogramming the TME to convert “cold” tumors into immunogenic “hot” landscapes.

**Methods:**

A literature search was conducted using PubMed, Scopus, and Google Scholar. The review was performed in a narrative and non‐systematic manner, focusing on studies addressing nanotechnology‐enhanced cancer immunotherapy.

**Results:**

We dissect the physicochemical and functional versatility of NPs, emphasizing size‐, charge‐, and ligand‐dependent strategies to enhance lymph node targeting, APC activation, and sustained cargo release. Innovations in metallic, lipid‐based, and biomimetic NPs are highlighted, including gold and lipid‐based NPs for enhanced immune responses. Furthermore, we explore combinatorial approaches, such as NP‐mediated co‐delivery of checkpoint inhibitors and chemotherapeutics, which amplify cytotoxic T‐cell responses and mitigate systemic toxicity. Clinical advancements, including Nab‐Paclitaxel and mRNA‐loaded lipid NPs, underscore the translational potential of these platforms, with trials demonstrating improved survival and manageable adverse profiles.

**Conclusion:**

However, challenges persist in optimizing targeting precision, scalability, and long‐term safety. Integrating breakthroughs in material science, immunology, and bioengineering, this review charts a roadmap for next‐generation nano‐immunotherapies, advocating patient‐specific designs and multimodal regimens. As the field strides toward clinical maturity, nanotechnology is poised to unlock the full potential of immunotherapy, paving the way for adaptive, immune‐guided, and potentially curative cancer therapies.

## Graphical abstract

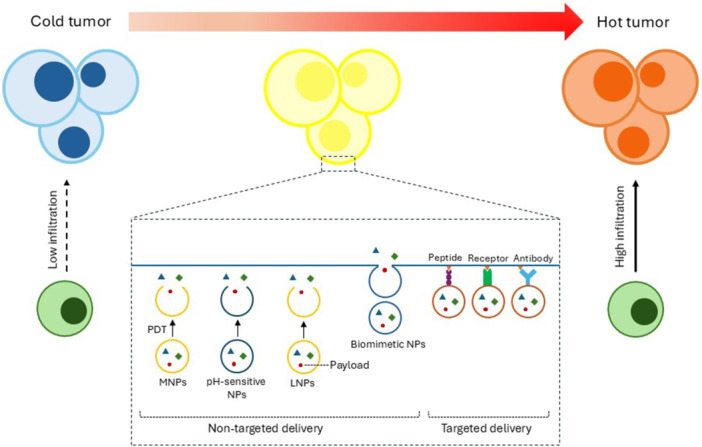

## 1 Introduction

Conventional cancer therapies, for example, surgery, radiotherapy, and chemotherapy, face limitations such as severe side effects, high recurrence risk, and restricted efficacy, driving the development of advanced approaches like immunotherapy [1, 2]. Immunotherapy, including passive strategies, for example, antibodies and cytokines, and active approaches, for example, cell‐based immunotherapy and cancer vaccine, shows promise in reducing metastasis and recurrence but faces challenges like autoimmune risks [3, 4]. Its efficacy is further limited in solid tumors due to the immunosuppressive tumor microenvironment (TME) hindering immune cell infiltration and the activation of oncogenic signaling pathways that drive tumor aggressiveness [5, 6, 7, 8].

Nanomedicine addresses these challenges using nanoparticles (NPs), which exploit the enhanced permeability and retention (EPR) effect for tumor accumulation, improve drug delivery, reduce systemic toxicity, and enhance bioavailability [9, 10, 11]. NPs can be functionalized with targeting moieties (antibodies, peptides, aptamers) to enhance specificity, mitigate drug resistance, and minimize off‐target effects [12, 13, 14, 15]. They also modulate immune responses, recruit to lymphoid organs, and enable theranostics [16, 17, 18].

Despite their potential, clinical translation hurdles persist, including optimizing targeting efficiency and understanding TME‐immune interactions [19, 20]. The current review focuses on refining NP design to improve biomaterial delivery, TME modulation, and diagnostic integration, positioning nanotechnology as a transformative tool in precision oncology [21, 22].

This study was conducted as a narrative review. A non‐systematic literature search was performed using PubMed, Scopus, and Google Scholar. The search strategy included keywords such as “nanoparticles,” “cancer immunotherapy,” “tumor microenvironment,” and “immune checkpoint.” Relevant studies were selected based on their focus on the physicochemical design of NPs, their immunomodulatory effects, and clinical applications in cancer immunotherapy.

### 1.1 Cancer Immunotherapies Influenced by Nanotechnology

Cancer immunotherapy primes the natural potencies of the host immune responses to identify, scavenge, and effectively clear tumoral cells. These enhanced anti‐cancer immune responses leverage the systemic surveillance ability of immune cells and bring about lingering immune memory, thus contributing to robust eradication of distant and remote metastasis and hindering the risk of recurrence. Numerous immunotherapeutic strategies are under active clinical and preclinical investigation [23, 24]. The primary goal of these modalities, like adoptive cell therapy, is to promote the quantity and functionality of tumor‐specific cytotoxic T lymphocytes (CTLs) in TME [25].

Dendritic cell (DC)‐based DNA vaccine utilizes plasmid DNAs encoding tumor‐specific antigens (TSAs)/tumor‐associated antigens (TAAs) and transfects them into DCs ex vivo or directly administers the synthetic tumor‐emulating peptides into the patient's circulation and activates resident antigen‐presenting cells (APCs) in the lymph tissues. These prompt APCs to participate in educating and boosting the cellular immune responses against TSAs and TAAs in vivo [26, 27, 28]. However, the high first‐pass metabolism and rapid degradation of these synthetic peptides in circulation reduce the amount that reaches APCs, thereby limiting the efficacy of cancer vaccines [29].

Checkpoint inhibitors (CPIs) modulate the interaction between tumor cells and T lymphocytes [30]. Immune checkpoints are inhibitory signals that maintain immunologic self‐tolerance by suppressing T‐cell activity. CPIs, such as cytotoxic T‐lymphocyte antigen‐4 (CTLA‐4) and programmed cell death‐1 (PD‐1) antibodies, block these suppressive pathways and restore the activity of tumor‐specific T cells that have been functionally exhausted by tumor cells [31]. In particular, CTLA‐4 expressed on T cells interacts with the surface antigens CD80 (B7‐1) or CD86 (B7‐2) on APCs with high affinity, thereby preventing conventional T‐cell activation through CD28 signaling [32]. Similarly, PD‐1 binding to PD‐1 ligand (PD‐L1) is considered to play a role in the disruption of T lymphocytes/APCs interaction, CTL's functional depletion, and accelerated regulatory T lymphocyte migration to the TME [33]. The United States Food and Drug Administration (FDA) approved these therapies for melanoma, non–small cell lung cancer (NSCLC), renal cancer, Hodgkin's lymphoma, bladder cancer, and head and neck cancer [34].

Also, Ab‐mediated cancer therapy methods include Ab‐mediated TAA/TSA targeting, conjugation of tumor‐targeted Abs with chemotherapy medications, and dual‐functioning Abs, which engage with the tumor cell and T lymphocyte to enhance CTL's attack on cancer [35, 36].

Despite the revolutionary impact of immunotherapy in the clinic, experimental documents disclose that only one out of five patients responds to currently approved immunotherapeutic agents; for example, about 12%–35% of the patients respond to CPIs [37]. Immune‐related adverse effects such as dermatitis, colitis, hepatitis, pneumonitis, and cytokine storm further impede their potencies [38]. Cold tumors (non‐immunogenic) with depleted amounts of T cells or downregulated PD‐L1 on cancerous cells are highly resistant to CPI‐mediated immunotherapies [39]. For instance, developing more efficient delivery routes to transfer TSA/TAA to APCs and designing more promising adjuvants to stimulate anti‐tumor immunity, accompanied by increasing the sensitivity of the TME to immunotherapies [40, 41].

While modern immunotherapies have opened a new era in cancer treatment, low response rates, immune‐related toxicities, and resistance in “cold” tumors reveal the dire need to develop an advanced delivery strategy. Nanotechnology provides transformative opportunities: protecting antigen presentation from degradation, allowing for targeted delivery to immune cells, and remodeling the TME to turn non‐immunogenic into immunoresponsive tumors. Hence, nanotechnology bridges conventional immunotherapeutics to next‐generation precision medicine and forms the basis for nano‐engineered cancer immunotherapies discussed in further sections.

### 1.2 Fundamentals of NPs in Cancer Immunotherapy

**Figure 1:**
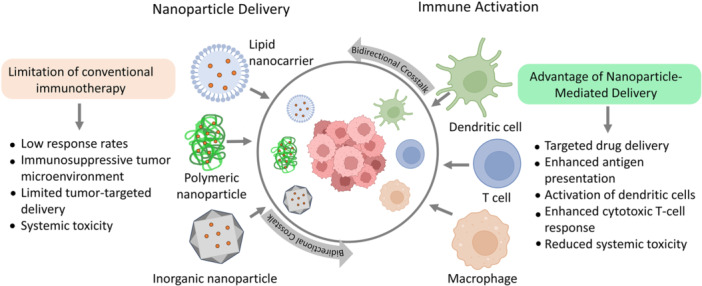
Schematic representation of the bidirectional crosstalk between nanoparticle‐based platforms and immune cells in cancer immunotherapy. Different nanoplatforms, including inorganic (metallic), lipid‐based, and polymeric nanoparticles, interact with key immune cells to enhance antigen presentation and stimulate antitumor immune responses within the tumor microenvironment.

NPs have gained prominence over recent decades due to their unique physicochemical traits, including tunable size, high surface‐to‐volume ratio, flexibility for chemical modification, and capacity for targeted delivery and controlled release of encapsulated components [42, 43]. These properties enable NPs to modulate the immune response by delivering TAAs and immunogenic molecules directly to immune cells, thereby promoting cancer‐specific cytotoxicity and converting non‐immunogenic (“cold”) tumors into immunologically active (“hot”) phenotypes through enhanced T‐cell recruitment within TME [44, 45]. A schematic overview of the bidirectional crosstalk between NP‐based platforms and immune cells in cancer immunotherapy is shown in Figure 1.

On the other hand, NPs′ capability to leak from most physiologic barriers, such as the blood‐brain barrier, and transfer immunotherapeutic agents, makes NPs the primary option for chemotherapy, photodynamic therapy [46], hyperthermia therapy, and other image‐guided cancer treatments of solid cancers. Collectively, NPs have demonstrated improved survival and durable immune responses in preclinical models [47].

The selective accumulation of NPs in tumors was first observed in the 1980s, leading to the identification of the EPR effect, which describes how leaky tumor vasculature and defective lymphatic drainage enable passive NP accumulation within the TME [48]. Building upon this principle, surface functionalization of NPs with specific ligands such as antibodies, peptides, or albumin has been shown to improve biodistribution, active targeting, and immune cell interaction [49]. For instance, albumin‐coated platinum (Pt) NPs exploit the tumor‐associated protein Secreted Protein Acidic and Rich in Cysteine (SPARC) to enhance selective uptake by cancer cells through the EPR‐mediated mechanism [50, 51].

NPs with diameters ranging from 10 to 100 nm are generally considered optimal for cancer therapy. Particles smaller than 10 nm are rapidly cleared through renal excretion, whereas those larger than 100 nm may face limited tissue penetration and uncertain biodistribution [52, 53]. Nano‐based drug delivery systems currently encompass an immense list of nanocarrier devices with specific modulatory effects on certain aspects of the immunity–cancer interactions [54]. The design of polymeric nanoformulations, such as poly lactic‐co‐glycolic acid (PLGA) lipids and polyethylene glycol (PEG), is a milestone of immune‐oncology, and these are commonly exploited in a variety of FDA‐approved nanodelivery systems because of their marked biocompatibility. Furthermore, metal NPs, liposomes, micelles, dendrimers, and artificial exosomes are widely used in synthesizing nano‐based drug delivery systems, primarily because of their efficient localization and low systemic toxicity [55, 56, 57]. In summary, the EPR effect and surface modification strategies represent fundamental mechanisms that underpin the tumor selectivity and immune‐modulatory potential of NP‐based cancer immunotherapy.

## 2 Prominent Properties of NPs Leveraging the Cancer Immunotherapeutic Potencies

### 2.1 Effect of Physicochemical Properties on the In Vivo Fate

Convenient anti‐cancer drug delivery routes are challenging because of the premature destruction of tumor antigens and other antigenic peptides and insufficient immune stimulation [48]. The pharmacodynamics and pharmacokinetics of the administered NPs are attributed to their size, elasticity, surface properties, and charge [58]. The influence of NP size and morphology on biodistribution has been extensively studied. The passive accumulation of NPs in tumors through the EPR effect has been described in Section Methods, 3; here, we focus on how size and other physicochemical features govern lymphatic trafficking and immune targeting. NP synthesis can be tailored for specific applications by adjusting their biophysical properties. For example, efficient lymph node targeting requires an optimal particle size, surface area, and hydrophobicity [59]. The NP diameter orchestrates their circulation and internalization behavior. Small NPs (< 10 nm) are prone to prominent extravasation (i.e., renal excretion), while large NPs (> 110 nm) face early entrapment within the lymphatic systems [60]. However, moderate NPs (10–110 nm) have an extended circulation time and efficiently localize in the lymph nodes [61]. Consistently, Reddy et al. [62] witnessed that 25–50 nm polypropylene sulfide NP carriers (PPS‐NPs) drained into lymph nodes in 5 days following application, and about 47% of PPS‐NPs were internalized into nodal APCs. In the same line, Wan et al. [63] demonstrated that, upon intradermal administration of 20 and 120 nm NPs, the former NPs exhibited strong accumulation in the lymphatic system, while the latter exerted significantly lower delivery rates (up to 10%).

The morphology of NPs is associated with their trafficking and localization in the target area [64]. On the other hand, NPs′ charge is closely related to their cellular absorption. For instance, cationic NPs contribute to a more pronounced immune response compared to neutral and anionic NPs. Although the engulfment of positively charged NPs is easier for local APCs, cationic NPs are significantly less mobile due to binding to negatively charged extracellular milieu components, which dampens their tissue permeability [65]. Indeed, the administration of cationic NPs into systemic circulation is challenging due to the risk of hemolysis induction, platelet aggregation, and other systemic adverse effects [66] due to premature cargo leakage and alterations in circulation kinetics and internalization [67]. Another study by Pourgholi et al. [68] disclosed that spatially balanced NPs (e.g., PEG polymers) with modestly negative or positive surface charges show a minimized propensity to non‐specific bindings. Macrophage engulfment is induced to scavenge NPs by providing a greater positive or negative surface charge. Considering the role of steric stability and surface charge of NPs in their design will help achieve more specific targeting.

### 2.2 Engineering the Synthesized NPs to Boost Their Efficiency

Engineering NPs can significantly enhance targeting efficiency, delivery performance, and immunomodulatory interactions [69]. As discussed in Section Methods, 3, surface modification plays a central role in improving NP functionality; here, we focus on how engineering approaches such as controlled release design and ligand‐mediated targeting can further optimize therapeutic outcomes. Functionalized NPs improve biocompatibility, in vivo stability, and drug solubility, extend circulation half‐life, enhance tumor localization, and minimize premature drug release, thereby enabling efficient multimodal cancer immunotherapy [70, 71]. NPs could be constructed to accumulate within the cell and release their cargo depending on the physicochemical alterations in the target environment. These changes include intrinsic factors such as changes in pH, redox potential, and enzymes [72, 73] or external interventions, such as optical, thermal, and magnetic stimuli [74].

Thus, the therapeutic effect occurs at the desired location in the intended condition. Controlled release not only improves the response rates of drugs but also reduces systemic side effects, including a cytokine storm [75, 76]. This is of interest in the case of immunotherapies with a small therapeutic window. The efficiency of NP internalization can be improved by engineering active transport mechanisms and ligand‐mediated recognition, such as mannose modification, which enhances lymph node targeting [77]. Synthetic NPs functionalized with TSA/TAA tune the tumor targeting of drugs. In this realm, dextran‐coated liposomes carrying ovalbumen (OVA) exert pH‐sensitive DC‐targeting in the TME, boosting cell‐mediated immunity through upregulation of major histocompatibility complex‐I (MHC‐I) on the cell surface. Co‐delivery of APC‐associated adjuvants that upregulate the B‐7.1 and B‐7.2 antigens on the APC surface profoundly improves the efficacy of the drug [78, 79].

### 2.3 NP Modulability

NPs hold great promise as carriers as they can carry a broad array of cargoes, including chemotherapeutic agents, small molecules, aptamers, peptides, nucleic acids, and assembling agents, such as lipids and polymers. Intriguingly, NPs transfer a disproportionately huge load of cargo [80, 81]. To shed light on this, a nanodisc with a 75 nm diameter could carry over 2000 small interfering RNA (siRNA). The significant entrapment efficiency of NPs is put into context by considering the Ab conjugate potency to transfer less than 10 siRNA [82].

Multiple drug resistance (MDR) proteins are well‐established efflux transporters on tumor cells, lowering the concentration of therapeutic components (e.g., Doxorubicin [DOX]) within the cell. Nanodiscs have been shown to block the MDR proteins. For example, P‐glycoprotein (Pgp) pumps the cytotoxic drug out of the cell, but NPs enter cancer cells by endocytosis, which helps them evade Pgp [83]. Interestingly, unlike other drug delivery systems, the physicochemical features and amount of their cargoes have minimal effect on NPs′ pharmacodynamics and pharmacokinetics as they are encapsulated within the construct [84, 85].

Beyond passive delivery, NPs can also be engineered for active targeting by incorporating ligands that recognize overexpressed tumor receptors, such as the transferrin receptor [86]. The high surface‐to‐volume ratio of NPs allows dense ligand presentation, which compensates for low individual binding affinities and promotes strong overall avidity [69]. These design strategies collectively enhance tumor selectivity and improve therapeutic accumulation at disease sites. Altogether, the fundamental physicochemical and engineering principles governing NP design form the basis for their success in cancer immunotherapy. Parameters such as size, charge, morphology, and surface functionalization dictate biodistribution, immune cell engagement, and therapeutic outcomes. By integrating controlled release mechanisms and ligand‐mediated targeting, NPs can achieve precise spatiotemporal delivery while minimizing systemic toxicity. These foundational insights guide the rational design of next‐generation nano‐immunotherapeutics with improved selectivity, efficacy, and translational potential.

### 2.4 Immunotherapeutic NP Types

**Figure 2:**
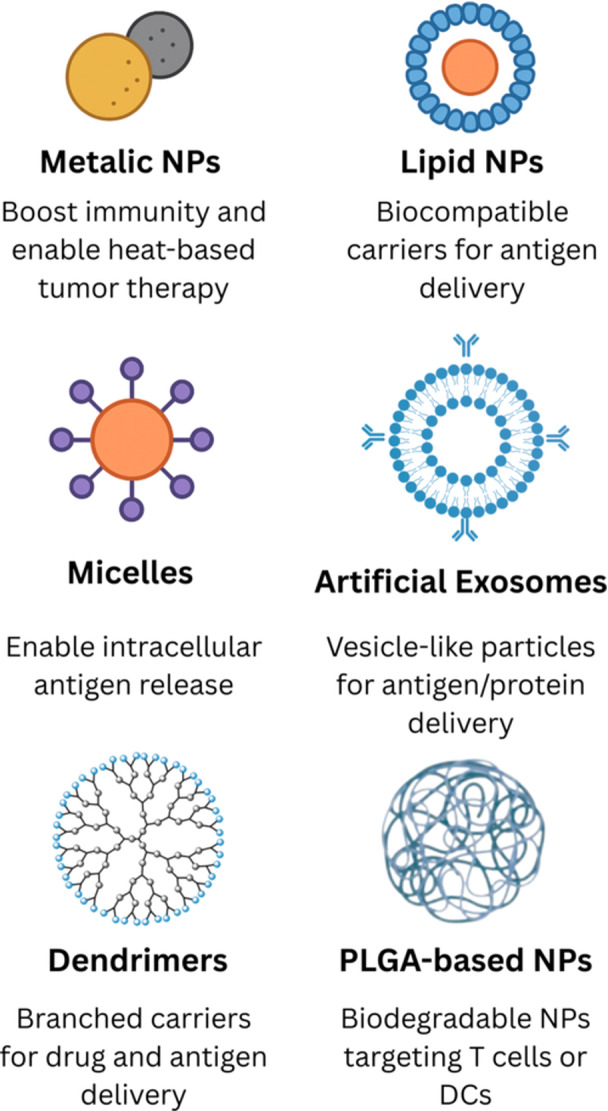
Main types of nanoparticles used in cancer immunotherapy.

NPs are commonly categorized as inorganic and organic NPs, according to their constituents. Given the vast network of immune interactions and TSAs/TAAs, NPs involved in cancer immunotherapy currently comprise an immense library of therapeutic agents [25]. Among dozens of nanodelivery systems, gold NPs (AuNPs), iron oxide NPs, PLGA NPs, liposomes, micelles, dendrimers, and artificial exosomes are broadly utilized in immunotherapy (Figure 2) [83, 84]. Notably, recent preclinical evidence has demonstrated the enhanced efficacy of combinational nano‐immunotherapy approaches in improving antitumor immune responses [87].

### 2.5 Metallic NPs (MNPs)

Investigations on MNPs interplay with the host immunity have become the focus of interest because of the recent fascinating outcomes in immunotherapy [46]. MNPs show higher uptake rates into the intracellular compartment than biodegradable nanodiscs. Recent investigations showed that NPs constituting Fe₃O₄ core and ZnO₂ shell and functionalized by specific DC ligands internalize strongly without the application of toxic transfection components [88]. Also, MNPs with gold shells are dis[68closed to penetrate tumor cells more readily than adjacent tissue due to the electrostatic force of gold‐coated MNPs and the surface charge of tumor cells [67]. A whole body of evidence has shown that delivery of CpG, a commonly used toll‐like receptor (TLR) 9 agonist adjuvant that emulates bacterial DNA, via MNPs accelerates Th lymphocyte activation, triggering CTL responses and proinflammatory cytokine release [89]. Almeida et al. [90] demonstrated that local treatment with 15 nm AuNP‐CpG encapsulated with OVA antigens resulted in a substantial systemic increase of IgG2a Ab titers and increased T lymphocyte activity, leading to impeded tumor growth and improved survival in a lymphoma.

MNPs, particularly gold NPs (AuNPs), exhibit intrinsic immunostimulatory properties that enhance anti‐tumor immunity. Lee et al. [91] demonstrated that 10 nm AuNPs maximally boost IgG antibody secretion in B cells by upregulating B‐lymphocyte‐induced maturation protein 1 (Blimp1) and suppressing paired box 5 (Pax5). Similarly, Almeida et al. [90] showed AuNPs conjugated with OVA (AuNP‐OVA) induced antigen‐specific T‐cell responses and prolonged survival in B16‐OVA tumor models, attributed to proinflammatory cytokine upregulation in DCs. These findings underscore AuNPs′ inherent adjuvant potential, warranting further exploration [90]. In addition, MNPs can counteract MDR by interacting with glutathione (GSH). Zeng et al. [50] developed daunorubicin‐loaded Pt‐NPs that reduced the drug resistance index from 12.4 to 7.9 in resistant tumors via GSH depletion and dual activation of caspase‐8 and caspase‐9 apoptotic pathways, highlighting MNPs′ role in overcoming chemoresistance.

MNPs are pivotal in nanotheranostics, particularly in photothermal therapy (PTT). Gold, copper, and silver NPs have demonstrated multifunctional roles in cancer imaging and therapy, with copper nanoclusters emerging as efficient, low‐cost, and biocompatible theranostic agents for imaging and treatment applications [92], and polymer‐coated AgNPs showing sustained drug release and potent anticancer efficacy [93]. Gold nanoshells and cuprous oxide NPs absorb specific laser wavelengths, generating localized heat that destroys tumors while sparing healthy tissue. This thermal ablation releases TAAs and damage‐associated molecular patterns, promoting DC‐mediated T‐cell activation and systemic anti‐tumor immunity, including abscopal effects [94, 95, 96, 97]. Moreover, externally triggered thermoelectric and piezoelectric nanoplatforms have recently been reported to combine catalytic ROS generation and immune activation under magnetic or ultrasonic stimulation, offering precise spatiotemporal control of synergistic immunotherapy [98, 99, 100, 101]. Beyond their direct photothermal and catalytic functions, MNPs have also been engineered as immunomodulatory platforms. Likewise, macrophage‐targeted nanomedicines have shown promise in reshaping the immunosuppressive TME and enhancing the efficacy of cancer immunotherapy [102]. Similarly, TME modulation has been recognized as a crucial determinant of immunotherapy response, with emerging strategies aiming to reprogram “cold” tumors into immunologically active “hot” phenotypes [103]. To enhance targeting, MNPs are functionalized with biocompatible coatings: dextran‐coated MNPs with MHC‐Ig dimers and anti‐CD28 antibodies recruit CTLs to immunosuppressive TMEs [104], while anti‐PD‐1 antibody‐conjugated CAR‐T cells magnetically guided to tumors synergize with checkpoint blockade and cell therapy [49]. Tumor‐necrosis factor (TNF)‐α‐coated AuNPs further amplify immune activation in metastatic models [105].

Beyond PTT, AuNPs serve as versatile drug carriers. Au‐nanospheres (15–50 nm) minimize toxicity and enhance CT imaging, while bare gold nanorods modulate TLRs, NOD‐like receptors, and MAP kinases to suppress angiogenesis and activate inflammasomes, elevating proinflammatory cytokines [106, 107, 108]. Certain MNPs, as well as heavy metal ions like cadmium, can activate innate immune responses through inflammasome pathways, leading to the release of proinflammatory cytokines [109]. Functionalization with α‐PDL1 enables image‐guided immunotherapy [110]. Iron oxide NPs, like Ferumoxytol, polarize macrophages and induce caspase‐3‐mediated apoptosis, while branched PEI‐coated SPIONs enhance Th1 responses under UV‐β [111, 112, 113]. Mesoporous silica NPs, with tunable pores, deliver hGM‐CSF to double macrophage proliferation, exemplifying their capacity for controlled therapeutic release [114]. These advances underscore MNPs′ multifaceted roles in advancing immune‐oncology. MNPs have been considered one of the most versatile platforms in cancer immunotherapy due to their intrinsic immunostimulatory properties, multifunctional design, and theranostic possibilities [115]. Their capabilities, including photothermal ablation, antigen presentation enhancement, and overcoming drug resistance, make them highly valuable for both activating immune responses and targeting tumor destruction. However, long‐term biosafety, potential accumulation toxicity, and complex surface modification requirements are significant concerns that have to be resolved before clinical translation.

### 2.6 Lipid NPs

Lipid‐based NPs are promising for targeted cancer therapy due to their biocompatibility, controlled release, and ability to encapsulate hydrophobic agents. Synthetic high‐density lipoprotein NPs (sHDL‐NPs), for instance, enhance drug delivery by prolonging circulation and localizing in the TME. Yan et al. [116] demonstrated that DOX‐loaded sHDL‐NPs co‐delivered with anti‐PD‐1 antibodies in CT26 colon carcinoma models increased CTL infiltration by sevenfold, upregulated interferon‐γ (IFN‐γ), and achieved complete remission in 80% of mice without cardiotoxicity. The immunogenicity of lipid NPs depends on their coatings: Pluronic, chitosan, or PEG‐PLA. Chitosan NPs (355 nm) exhibit high bioadhesiveness and enhance siRNA delivery, as shown by Bastaki et al. [117], who used PD‐L1‐STAT3 siRNA‐loaded chitosan NPs to suppress tumor growth via STAT3/PD‐L1 axis inhibition. In contrast, smaller PEG‐PLA NPs (~175 nm) show stealth properties but limited macrophage uptake, while Pluronic NPs stimulate monocytes to overexpress TNF‐α, interleukin (IL)−6, IL‐10, and IL‐12, suggesting adjuvant potential.

Immunoliposomes are engineered to enhance T cell activity against immunosuppressive tumors. Evans et al. [55] developed CD90‐targeted immunoliposomes encapsulating TGF‐β inhibitors, which boosted T cell activation, granzyme expression, and tumor suppression in mice. Lipid NPs also enable transient CAR mRNA delivery to T cells, reducing toxicity compared to electroporation [55]. Lim et al. [118] designed “tumosomes”, 100 nm liposomes loaded with tumor membrane proteins, monophosphoryl lipid A (MPLA), and dimethyl‐dioctadecyl ammonium, which enhanced antigen‐specific immunity and survival in murine models. PEGylated liposomes functionalized with anti‐CD40 and CpG oligonucleotides further concentrate therapy in the TME by activating TLR and CD40 pathways [53].

pH‐sensitive liposomes exploit the acidic TME for targeted cargo release. Encapsulated cyclic di‐GMP (a STING agonist) in fusogenic liposomes enables cytosolic delivery to activate IFN‐1 pathways. Dextran‐based liposomes modified with PEG and TGF‐β1 receptors are designed for enhancing CD8^+^ T cell infiltration [119]. Curdlan/mannan‐coated pH‐sensitive liposomes delivered antigens to DCs, eliciting stronger anti‐tumor responses than dextran variants. Cationic lipid adjuvants are highlighted in pH‐sensitive systems, which synergize with released immunotherapeutics to remodel the TME [120, 121]. These advances underscore lipid NPs′ versatility in balancing targeted delivery, immune activation, and microenvironment modulation for precision oncology. Overall, lipid‐based NPs stand out among clinically advanced nanoplatforms due to their high biocompatibility, ability to encapsulate a diverse range of biomolecules, and tunable release behavior. Their flexibility allows for efficient lymphatic delivery, immune cell activation, and synergy with checkpoint blockade or mRNA‐based approaches. Nevertheless, issues such as limited stability, potential off‐target immune activation, and rapid clearance by the mononuclear phagocyte system remain critical challenges that require further optimization for successful clinical translation.

## 3 Cell Membrane‐Camouflaged NPs

### 3.1 Micelles

There is a wide range of applications of micelles in cancer treatment as carriers for imaging, chemotherapy, radiotherapy, and immunotherapy. The synthesis of micelles is comparatively easier than that of other NPs. The biodegradability and nontoxicity of these formulations make them suitable for carrying therapeutic payloads. However, new studies show that the cytoplasmic delivery of antigens is possible using micelles as carriers. A pH‐responsive micelle composed of di‐lauroyl phosphatidylcholine and deoxycholic acid was synthesized both to deliver antigens to the cytoplasm and to induce an immune response [54]. Micelles were taken up by DCs mainly via macropinocytosis and delivered OVA into the cytosol. These micelles are helpful for increasing the capability of cellular immunity in the treatment of cancer. Polymeric micelles, which are self‐assembled structures, have been in cancer research for quite some time owing to high drug loading and the ability to modulate surface characteristics by simple chemistry [94, 95]. Polymeric micelles loaded with 6‐thioguanine have been studied to suppress the effect of myeloid‐derived suppressor cells (MDSCs) and to enhance anti‐tumor T cell response in a murine model [56]. MDSCs are responsible for downregulating the efficacy of anti‐tumor immunity and are supposed to be a significant hindrance to therapy. The cationic polymer, such as polyethyleneimine (PEI), has a property of inducing necrosis by recruiting inflammatory cytokines at the site of the tumor. PEI‐polymeric micelles were composed of cationic charge‐masking HA to overcome the necrotic effect. Thus, once the HA‐PEI micelle is internalized into the cell, it sheds off the HA layer, revealing the cationic complex in the cytosol of the cell that leads to the selective production of cytokines like monocyte chemoattractant protein‐1 and TNF‐α, further inhibiting cell growth [122, 123].

Peng et al. [125] showed the stimulation of the immune response using micelles with the combined action of PTT with immunotherapy. The immune response can be stimulated by regulating metabolism‐related enzymes. Due to the accumulation of IR780 in the tumor, followed by migration to the lymph node, PTT can be performed, resulting in the inhibition of indoleamine 2,3‐dioxygenase (IDO). The inhibition of IDO leads to the activation of T lymphocytes, followed by the inhibition of distal tumor growth (abscopal effect). The combined therapy of this micellar system kills the tumor by PTT and inhibits distal tumor growth post‐PTT in vivo in BALB/c‐nu mice [124]. Polymeric micelles are currently considered to be a more exploratory NP carrier system for cancer immunotherapy. The delivery of tyrosinase‐related protein two peptide antigen and adjuvant to the lymph node in the B16F0 melanoma mouse model was performed using cationic diblock polymeric micelles. The resulting increased T lymphocyte anti‐cancer activity indicates the efficiency of these kinds of micelles in treating cancer by improving the immune response [125]. Micelle‐based nanocarriers have demonstrated versatile functionality in cancer immunotherapy due to their simple synthesis, biodegradable nature, and capability of co‐delivering antigens, adjuvants, and therapeutic agents with controlled release. Their tunable surface chemistry could enable further antigen presentation, T cell activation, and synergistic effects in combination with photothermal or metabolic enzyme inhibition. Despite this strong potential, reaching full clinical utility requires overcoming some challenges, like limited stability in vivo, premature drug release, and poor targeted lymphatic accumulation.

### 3.2 Artificial Exosomes

Artificial exosomes are biomimetic vesicles engineered to mimic natural exosomes and are increasingly explored as nanocarriers for delivering therapeutic proteins and antigens [126, 127]. The use of biomimetic exosomes for the delivery of cargo for immunotherapy has gained more attention. Delivery of the monoclonal Ab DEC205 to DCs can be performed using biomimetic exosomes. The synthesis of these exosomes is similar to that of liposomes. In the first study, tumor antigens were delivered to DCs for immunotherapy. The synthesis of artificial exosomes is very easy compared to other carrier systems, and this treatment method could be a key to future nanomedicine for immunotherapy. Artificial exosomes can be modified using MHC class I peptides and liposomes for DC targeting and T cell activation. The in vivo studies showed increased T cell activation by the action of MHC class I peptide delivery to DCs. The liposome peptide containing artificial exosomes was found to be very stable and suitable for targeting DCs [128, 129, 130]. Artificial exosomes are a rapidly emerging class of biomimetic nanocarriers capable of precise antigen and protein delivery to APCs, effectively bridging natural immune communication with engineered precision [131]. Their excellent biocompatibility, stability, and promotion of DC activation and T cell priming provide a strong impetus for personalized cancer immunotherapy applications. However, challenges associated with large‐scale manufacturing, batch variability, and a short circulation half‐life represent major barriers for artificial exosomes in clinical applications.

### 3.3 Dendrimers

Dendrimers are used to deliver OVA to immune cells by incorporating OVA into guanidine‐terminated dendrimers by utilizing the helix B region of OVA. Immunodendrimers are used to treat ovarian cancer in BALB/c mice. A half‐generation poly(propyl imine) dendrimer is conjugated with an immunotherapeutic Ab and loaded with the anti‐cancer drug paclitaxel. This immunodendrimer significantly reduced systemic toxicity and tumor volume, demonstrating the efficacy of dendrimers as a carrier vehicle for both therapeutic drugs and antigens [132]. Dendrimers represent a versatile and highly tunable nanoplatform for the simultaneous delivery of therapeutic agents and immunogenic antigens. Their branched architecture allows for precise control of size, charge, and surface functionality for efficient cellular uptake with reduced systemic toxicity. However, biodegradability concerns, potential cytotoxicity at high generation, and complex synthesis processes remain a challenge toward their large‐scale clinical translation.

### 3.4 Modified PLGA‐Based NPs

PLGA‐based NPs functionalized with immune‐targeting ligands enhance T cell‐mediated anti‐tumor responses. Schmid et al. developed FDA‐approved PLGA‐PEG NPs conjugated with CD8α Fab fragments to specifically target CD8α^+^ T cells in tumors, blood, and lymphoid organs, improving T cell activation over systemic drug administration. Further modification with PD‐1 antibodies (PD‐1‐PLGA‐PEG NPs) enabled co‐delivery of TGFβ inhibitors (SD‐208) and TLR7/8 agonists (R848), achieving sustained drug release, reduced off‐target toxicity, and prolonged survival in colorectal tumor‐bearing mice. These NPs enhanced tumor‐specific T lymphocyte recruitment and payload delivery, offering a safer, off‐the‐shelf strategy to overcome immunotherapy limitations [133]. Given that tumor progression is influenced not only by biochemical cues but also by physical properties of the TME, such as extracellular matrix stiffness, integrating responsiveness to these mechanical factors into NP design could further enhance therapeutic efficacy [134].

PLGA NPs also excel in DC targeting to amplify antigen presentation and anti‐tumor immunity. Surface modification with CD205‐ or CD40‐specific antibodies improves DC uptake, antigen delivery, and IL‐10 production, fostering prolonged survival in preclinical models [135, 136]. Combining PLGA NPs with PTT, via gold nanoshells and anti‐PD‐1 peptides, enables localized tumor ablation, PD‐1/PD‐L1 blockade, and systemic immune activation against metastases [137]. In addition, polymeric matrices like hydrogels show promise for sustained cytokine release (e.g., IL‐2, IFN‐α), though protein denaturation during encapsulation necessitates optimization for clinical translation. These strategies underscore PLGA NPs′ versatility in balancing targeted delivery, immune activation, and combination therapies [138]. Among all polymeric delivery systems, PLGA‐based NPs represent one of the most clinically advanced platforms in cancer immunotherapy due to their biocompatibility, controlled release properties, and flexibility for surface functionalization. Their ability to co‐deliver immunomodulators, CPIs, and cytokines enables precise immune activation with reduced systemic toxicity. However, to fully realize their translational and clinical potential, several challenges must be addressed, including protein instability during encapsulation, limited mechanical responsiveness to the TME, and scalability issues.

## 4 NP‐Engineered Cancer Vaccines

NPs are potent tools for delivering TAAs/TSAs to DCs, enabling innate immune activation via TLR‐mediated pathways. Heo et al. [139] designed PLGA NPs co‐encapsulating STAT3‐specific siRNA and the TLR7 agonist R837, which internalized into DCs, activated TLR7 signaling, and suppressed immunosuppressive genes, inhibiting tumor growth. Similarly, Cubillos‐Ruiz et al. [140] developed linear PEI‐based micelles encapsulating siRNA, which were selectively taken up by immunosuppressive CD11c^+^PD‐L1^+^ DCs in ovarian tumors. PEI‐siRNA transformed these DCs into immunostimulatory APCs via TLR5/7 activation, enhancing T cell responses. These platforms highlight NPs′ ability to reprogram immunosuppressive microenvironments while minimizing off‐target effects. Clinical advancements include DepoVax (DPX‐0907), a liposomal DC vaccine loaded with seven tumor epitopes, which traps antigens in DCs and activates CTLs in phase I trials for breast, ovarian, and prostate cancers [141]. Theranostic NPs, such as iron oxide–zinc oxide core–shell NPs, simultaneously deliver antigens and imaging agents to DCs, enabling real‐time tracking and immune activation [142], while upconversion NPs (UCNPs) complexed with OVA enhance CD8⁺ CTL proliferation and allow optical tracking of migratory DCs [143].

NP‐based vaccines enhance antigen‐specific T cell responses by targeting APCs in lymphoid tissues, leveraging immunostimulants like CpG oligonucleotides. CpG activates DCs via TLR9, promoting CD8^+^ CTLs, NK cells, and Th1 responses that synergize with checkpoint blockade therapies [144]. Nano‐engineered DC vaccines, such as PLGA NPs loaded with antigens and adjuvants, sustain antigen release and prevent tumor relapse post‐surgery. For example, anti‐PD‐1 peptide‐loaded PLGA NPs combined with gold nanoshells achieve photothermal ablation at primary tumor sites while inducing systemic immunity via PD‐1/PD‐L1 blockade [145]. In addition, polymeric NPs encapsulating cytokines (e.g., IL‐12, IFN‐α) or theranostic platforms like UCNP‐antigen complexes optimize DC maturation and CTL activation, though challenges like protein denaturation during encapsulation require refinement [146]. Identifying immune‐related biomarkers like Netrin‐1 helps stratify patients for tailored immunotherapy, as varying levels of immune infiltration strongly influence therapeutic outcomes in solid tumors [147]. Within immuno‐oncology, NP‐engineered cancer vaccines represent a clinically promising approach by marrying precise antigen delivery with immune system modulation. Capable of co‐delivering TAAs, adjuvants, and immunomodulators, NPs ensure strong and durable T cell activation with minimum systemic toxicity. Despite significant progress, challenges such as antigen instability during formulation, variability in patient immune profiles, and the need for scalable, clinically compliant manufacturing remain major barriers to achieving consistent clinical translation.

### 4.1 Clinical Trials of NP‐Based Applications in Cancer Immunotherapy

NP albumin‐bound paclitaxel (Nab‐Paclitaxel) is a 130 nm particle formulated via non‐covalent bonding between paclitaxel and human serum albumin, enhancing tumor‐targeted delivery 1. Its mechanism leverages albumin's binding to endothelial gp60 receptors and SPARC (secreted protein acidic and rich in cysteine) in the TME, promoting transcytosis and drug accumulation [148, 149]. Compared to solvent‐based paclitaxel, Nab‐Paclitaxel reduces hypersensitivity reactions and improves tolerability [150]. Preclinical studies demonstrate its ability to inhibit mitosis and cell division, with high concentrations achieved in TME [151]. These properties underpin its broad application in clinical trials, particularly in breast and lung cancers, often combined with immunotherapy agents like CPIs or monoclonal antibodies (mAbs).

In metastatic breast cancer (MBC), Nab‐Paclitaxel combinations show promising efficacy. A phase II trial (NCT00629499) combining Nab‐Paclitaxel, cyclophosphamide, and trastuzumab in HER2^+^ patients reported 100% 24‐month disease‐free survival, with neutropenia (53% grade 3/4) as the primary adverse event [152]. Another study (NCT00110084) evaluating Nab‐Paclitaxel with gemcitabine in MBC demonstrated a 76% overall survival (OS) and 60% 6‐month progression‐free survival (PFS), with neutropenia (54%) and thrombocytopenia (12%) as common toxicities [153]. For HER2‐overexpressing MBC, a trial (NCT00093145) combining Nab‐Paclitaxel, carboplatin, and trastuzumab achieved a 62.5% overall response rate (ORR) and 81.3% clinical benefit, albeit with frequent neuropathy (63%) [153]. A phase II study (NCT00709761) pairing Nab‐Paclitaxel with lapatinib reported a 53% ORR but noted treatment discontinuation in 82% of patients due to adverse events, including diarrhea and febrile neutropenia [154].

In NSCLC, a phase II trial (NCT00553462) combining Nab‐Paclitaxel, carboplatin, erlotinib, and radiation reported a 59% ORR and 11‐month PFS, though dyspnea (64%) and fatigue (87%) were common. A MBC study (NCT00654836) testing Nab‐Paclitaxel, carboplatin, and bevacizumab showed a 16‐month PFS and 21‐month OS, with manageable hematologic toxicities [155]. Novel combinations, such as Nab‐Paclitaxel with PD‐1 inhibitors (e.g., NCT02752685, NCT04249167), are under investigation to enhance immune activation while minimizing systemic toxicity.

Nab‐Paclitaxel is increasingly paired with immune CPIs. For example, mRNA‐2416, a lipid NP encoding OX40L, is being tested with durvalumab (anti‐PD‐L1) in advanced malignancies (NCT03323398). Similarly, mRNA‐2752, encapsulating OX40L, IL‐23, and IL‐36γ mRNAs, is in phase I trials with durvalumab (NCT03739931). Other combinations include Cetuximab (EGFR inhibitor, NCT00736619), Pertuzumab/Trastuzumab (NCT01730833), and Atezolizumab (NCT04249167), aiming to synergize chemotherapy with immune modulation [156, 157]. These trials highlight efforts to leverage Nab‐Paclitaxel's delivery efficiency to enhance CPI efficacy while mitigating adverse events like neutropenia and neuropathy.

Emerging NP platforms include DOTAP‐cholesterol‐TUSC2 NPs (NCT01455389), which induce apoptosis in NSCLC by delivering the TUSC2 tumor suppressor gene [158]. GPX‐001, a lipid NP carrying TUSC2, is being evaluated with osimertinib (NCT04486833). pH‐sensitive polymeric NPs for Cetuximab delivery (NCT03774680) aim to reduce intestinal toxicity via targeted release 3. PRECIOUS‐01, a PLGA NP loaded with NY‐ESO‐1 antigen and iNKT activator IMM60, is in phase I trials for advanced solid tumors (NCT04751786) 9. In addition, Nab‐Rapamycin (ABI‐009), a 100 nm NP, is being tested with pazopanib in sarcomas (NCT03660930) [159]. These innovations underscore the expanding role of engineered NPs in improving drug targeting, reducing toxicity, and enhancing combinatorial immunotherapy efficacy [160]. The clinical trials related to Nab‐Paclitaxel and emerging NP platforms, including combinations with CPIs and targeted therapies, are summarized in Table 1. Clinical translation of NP‐based immunotherapies proceeds with rapid strides, with platforms such as Nab‐Paclitaxel and mRNA‐loaded lipid NPs leading toward regulatory approval. Taken together, these trials illustrate the potential of nanoscale formulations to improve drug solubility, enable selective delivery, and synergize with immune checkpoint blockade with reduced systemic toxicity. However, clinical results also underscore remaining challenges, including inter‐patient variability, manufacturing complexity, and limited long‐term safety evaluation [161, 162]. Bringing preclinical success to clinical consistency will require improved patient stratification, scalable GMP manufacturing, and incorporation of predictive biomarkers to optimize efficacy and safety across a range of cancer types [163, 164]. A comparison across these trials yields a number of important lessons that can be derived for the optimization of future nano‐immunotherapeutic designs. Indeed, combination regimens that incorporate NP formulations with CPIs or targeted agents tend to yield higher response rates but often at the cost of increased hematologic or immune‐related toxicity, emphasizing the need for precise dose scheduling and immune monitoring. Moreover, patient‐specific factors such as tumor burden, immune infiltration status, and prior treatment exposure critically influence clinical outcomes, therefore underlining the importance of biomarker‐based patient selection [165]. Whereas lipid‐based and polymeric NPs demonstrate favorable safety and efficiency of delivery, metallic and hybrid systems remain to be further validated because of their potential to accumulate and cause long‐term toxicity. These lessons taken together stress that the success of nano‐immunotherapy in clinics will not only depend on NP design but also on personalized integration with treatments and strategies of long‐term immune management [166].

**Table 1:** Summary of nanoparticle‐engineered cancer vaccine platforms, their immune mechanisms, therapeutic advantages, and key challenges.

NP platform	Representative example	Immune‐modulatory mechanism	Therapeutic advantage	Key challenges	References
PLGA NPs	PLGA NPs co‐encapsulating STAT3‐siRNA and TLR7 agonist R837	Activation of TLR7 signaling; suppression of immunosuppressive genes; DC maturation	Strong T cell activation and tumor growth inhibition	Protein instability during encapsulation; scalability issues	[139, 167]
PLGA–Au hybrid NPs	Anti‐PD‐1 peptide‐loaded PLGA NPs combined with gold nanoshells	PD‐1/PD‐L1 blockade; photothermal ablation; systemic immune activation	Synergistic immune response and tumor relapse prevention	Manufacturing complexity; potential overheating risk	[145]
PEI‐based micelles	Linear PEI–siRNA micelles targeting CD11c+PD‐L1+ DCs	TLR5/7‐mediated DC activation; reprogramming of tolerogenic DCs	Converts immunosuppressive DCs to a stimulatory phenotype; enhances CTL responses	Cytotoxicity at high concentrations; limited in vivo stability	[140, 168]
Liposomal Vaccine (DepoVax)	DPX‐0907 liposomal DC vaccine with seven tumor epitopes	Antigen entrapment and presentation by DCs; CTL priming	Proven safety in phase I trials; robust antigen‐specific responses	Complex GMP manufacturing; cold‐chain requirement	[141, 169]
Iron oxide–zno core–shell NPs	Theranostic NPs for DC targeting and antigen delivery	Dual antigen delivery, magnetic targeting, and imaging capability	Enables real‐time immune tracking and therapy monitoring	Metal accumulation: potential long‐term toxicity	[142]
Upconversion NPs (UCNPs)	UCNP–OVA complexes	Optical activation of immune signaling; CD8+ CTL proliferation	Real‐time antigen tracking; minimal photodamage	Low biodegradability; complex synthesis	[143]
CpG‐loaded polymeric NPs	PLGA/CpG nanovaccine	TLR9‐mediated DC activation; Th1 polarization	Potent CTL and NK cell activation; synergy with checkpoint blockade	Risk of systemic cytokine release; dosing control	[144, 170]

## 5 Conclusion and Future Perspectives

The integration of nanotechnology with immunotherapy represents a paradigm shift in cancer treatment, offering innovative solutions to long‐standing challenges in oncology. By leveraging the unique physicochemical properties of NPs, such as tunable size, surface functionalization, and stimuli‐responsive release, researchers have unlocked unprecedented precision in delivering immunotherapeutic agents, reprogramming the TME, and amplifying systemic anti‐tumor immunity. From MNPs enabling photothermal‐immune synergy to biomimetic systems evading immune clearance, this review underscores how engineered nanoplatforms enhance the efficacy of CPIs, cancer vaccines, and adoptive cell therapies while minimizing off‐target toxicity. Despite remarkable progress, critical hurdles remain. The heterogeneity of human tumors, variable NP biodistribution, and incomplete understanding of NP‐immune cell interactions necessitate further optimization of targeting strategies and predictive biomarkers. Scalability, long‐term safety, and regulatory frameworks for multifunctional NPs also demand rigorous evaluation to bridge the gap between preclinical promise and clinical reality. However, emerging advances in machine learning‐driven NP design, patient‐specific antigen profiling, and multimodal combination therapies indicate a future direction toward adaptable and evolution‐responsive nano‐immunotherapies. Addressing these challenges and advancing regulatory harmonization will be essential to fully translate nano‐immunotherapy innovations from bench to bedside. The clinical success of Nab‐Paclitaxel and mRNA lipid NPs exemplifies the transformative potential of this field, yet their full impact will depend on interdisciplinary collaboration among material scientists, immunologists, and clinicians. The integration of artificial intelligence and computational modeling is an emerging area that may enable the rapid prediction of biodistribution patterns and immune responses, thereby revolutionizing the design of NPs and the personalization of immunotherapy. As nano‐immunotherapies advance toward clinical translation, ethical considerations must be integrated into the translational frameworks, addressing equitable access, data privacy in patient‐specific models, and long‐term biosafety. As we venture into an era of precision oncology, the fusion of nanotechnology with immunotherapy will not only redefine therapeutic boundaries but also empower a new generation of curative treatments. By prioritizing patient‐centric design and robust translational pipelines, this synergy promises to turn the tide against cancer, transforming it from a lethal adversary into a manageable chronic condition. In contrast to previous reviews that have mainly provided general overviews of nanotechnology applications in immunotherapy, the present work uniquely emphasizes the mechanistic and translational dimensions of nano‐immunotherapy. It integrates recent advances in NP engineering with immunological mechanisms, highlighting how nanomaterials can reprogram the TME and synergize with checkpoint blockade, cancer vaccines, and adoptive cell therapies. Moreover, this review outlines ongoing clinical trials and FDA‐approved nanomedicines, offering a comprehensive and forward‐looking perspective for the development of next‐generation precision nano‐immunotherapies (Table 2).

**Table 2:** Clinical trials of the application of nanomedicine in immunotherapeutic and non‐immunotherapeutic cancer therapy.

NPs	Payload	Combination therapy status	Type of cancer	Status	Phase	No.	NCT number
DOTAP: Chol‐TUSC2 (cholesterol NPs)	*TUSC2* (tumor suppressor gene)	Tarceva (erlotinib hy drochloride), dexamethasone, diph enhydramine	Lung cancer	Active, not recruiting	I, II	57	NCT01455389
Nab paclitaxel (albumin‐bound paclitaxel) axel)	Paclitaxel	Cyclophosphamide, trastuzumab	Breast cancer	Completed	II	63	NCT00629499
Carboplatin‐nab paclitaxel	Paclitaxel carboplatin	HLX10 (anti‐PD‐1)	Squamous non–small cell lung cancer	Recruiting	III	516	NCT04033354
Paclitaxel albumin‐stabilized NP formulation	Paclitaxel	Gemcitabine hydrochloride, bevacizumab (anti–VEGF)	Breast cancer	Completed	II	50	NCT00662129
ABI‐007 (nab‐paclitaxel)	Paclitaxel	Carboplatin bevacizumab (anti–VEGF)	Breast cancer	Completed	II	32	NCT00654836
Albumin‐bound paclitaxel (abraxane; ABI‐007)	Paclitaxel	Carboplatin herceptin	Breast cancer	Completed	II	32	NCT00093145
Abraxane	Paclitaxel	Bevacizumab (anti–VEGF) carboplatin	Breast cancer	Completed	II	33	NCT00675259
Nab‐paclitaxel	Paclitaxel	Sintilimab (anti‐PD‐1)	Stage III of gastric cancer	Recruiting	I, II	38	NCT04781413
Albumin‐bound paclitaxel (abraxane)	Paclitaxel	Cetuximab (EGFR inhibitor)	Head and neck cancer	Completed	I	25	NCT00736619
Nab‐paclitaxel	Paclitaxel	Pertuzumab, trastuzumab	Breast cancer	Active, not recruiting	II	65	NCT01730833
mRNA‐2416 (lipid NPs)	mRNA encoding human OX40L	Durvalumab (anti‐PD‐L1)	Relapsed/refractory solid tumor malignancies or lymphoma Ovarian cancer	Recruiting	I, II	117	NCT03323398
TKM 080301 (lipid NPs)	siRNA against the PLK1 gene product	_	Colorectal cancer with hepatic metastasis Pancreatic cancer with hepatic metastasis Gastric cancer with hepatic metastasis Breast cancer with hepatic metastasis Ovarian cancer with hepatic metastasis	Completed	I	1	NCT01437007
PRECIOUS‐01 (PLGA‐NPs)	Invariant natural killer T cell (iNKT) activator threitolceramide‐6 (ThrCer6, IMM60), (NY‐ESO‐1) cancer‐testis antigen peptides		Advanced solid tumor	Recruiting	I	15	NCT04751786
Nab‐paclitaxel	Paclitaxel	Lapatinib	ErbB2‐overexpressing metastatic breast cancer	Completed	II	60	NCT00709761
Nab‐paclitaxel	Paclitaxel	Gemcitabine adavosertib	Metastatic pancreatic adenocarcinoma Stage III pancreatic cancer, AJCC v6 and v7 Stage IV pancreatic cancer, AJCC v6 and v7 Unresectable pancreatic carcinoma	Active, not recruiting	I, II	133	NCT02194829
INT‐1B3 (lipid NPs)	miRNA (miR‐193a‐3p)	_	Solid tumors	Recruiting	I	80	NCT04675996
TUSC2‐NPs (GPX‐001)	TUSC2	Osimertinib (EGFR tyrosine kinase inhibitor)	Non‐small cell lung carcinoma	Not yet recruiting	I, II	100	NCT04486833
mRNA‐2752 (Lipid NPs)	mRNAs encoding human OX40L, IL‐23, and IL‐36γ	Durvalumab	Dose escalation: relapsed/refractory solid tumor malignancies or lymphoma Dose expansion: triple negative breast cancer, head and neck squamous cell carcinoma, non‐Hodgkin lymphoma, and urothelial cancer	Recruiting	I	126	NCT03739931
Albumin‐bound rapamycin (ABI‐009; nab‐rapamycin)	Rapamycin	Pazopanib hydrochloride	Advanced soft tissue sarcoma Locally advanced soft tissue sarcoma Metastatic soft tissue sarcoma	Recruiting	I, II	57	NCT03660930
Nab‐paclitaxel	Paclitaxel	Pembrolizumab (anti‐PD1)	HER2^‐^ metastatic breast cancer	Recruiting	_	70	NCT02752685
PLD (PEGylated liposomal Doxorubicin)	Doxorubicin	Pembrolizumab (anti‐PD1)	Ovarian cancer Fallopian tube cancer Peritoneal cancer	Active, not recruiting	II	26	NCT02865811
Nab‐paclitaxel	Paclitaxel	Atezolizumab (anti‐PD‐L1)	Locally advanced or metastatic triple‐negative breast cancer	Active, not recruiting	I	5	NCT04249167

## Author Contributions

**Nima Javanmehr:** writing – original draft, writing – review and editing, resources, conceptualization, investigation. **Asal Moazzami Ashtyani:** writing – original draft, investigation. **Robabehbeygom Ghafelehbashi:** validation, supervision, writing – review and editing. **Fateme Mousavi:** writing – review and editing. **Hossein Teimouri:** writing – review and editing, conceptualization, supervision, project administration. All authors read and approved the final manuscript.

## Ethics Statement

The authors have nothing to report.

## Consent

The authors have nothing to report.

## Conflicts of Interest

The authors declare no conflicts of interest.

## Data Availability

Data sharing is not applicable to this article as no new data were created or analyzed in this study.
